# Succinyl Chitosan-Colistin Conjugates as Promising Drug Delivery Systems

**DOI:** 10.3390/ijms24010166

**Published:** 2022-12-22

**Authors:** Natallia V. Dubashynskaya, Anton N. Bokatyi, Anatoliy V. Dobrodumov, Igor V. Kudryavtsev, Andrey S. Trulioff, Artem A. Rubinstein, Arthur D. Aquino, Yaroslav A. Dubrovskii, Elena S. Knyazeva, Elena V. Demyanova, Yuliya A. Nashchekina, Yury A. Skorik

**Affiliations:** 1 Institute of Macromolecular Compounds of the Russian Academy of Sciences, Bolshoi VO 31, 199004 St. Petersburg, Russia; 2 Institute of Experimental Medicine, Akademika Pavlova 12, 197376 St. Petersburg, Russia; 3 Almazov National Medical Research Centre, Akkuratova 2, 197341 St. Petersburg, Russia; 4 State Research Institute of Highly Pure Biopreparations, Pudozhsakya 7, 197110 St Petersburg, Russia; 5 Institute of Cytology of the Russian Academy of Sciences, Tikhoretsky 4, 194064 St. Petersburg, Russia

**Keywords:** chitosan, succinyl chitosan, colistin, drug-polymer conjugates, drug delivery systems

## Abstract

The growth of microbial multidrug resistance is a problem in modern clinical medicine. Chemical modification of active pharmaceutical ingredients is an attractive strategy to improve their biopharmaceutical properties by increasing bioavailability and reducing drug toxicity. Conjugation of antimicrobial drugs with natural polysaccharides provides high efficiency of these systems due to targeted delivery, controlled drug release and reduced toxicity. This paper reports a two-step synthesis of colistin conjugates (CT) with succinyl chitosan (SucCS); first, we modified chitosan with succinyl anhydride to introduce a carboxyl function into the polymer molecule, which was then used for chemical grafting with amino groups of the peptide antibiotic CT using carbodiimide chemistry. The resulting polymeric delivery systems had a degree of substitution (DS) by CT of 3–8%, with conjugation efficiencies ranging from 54 to 100% and CT contents ranging from 130–318 μg/mg. The size of the obtained particles was 100–200 nm, and the ζ-potential varied from −22 to −28 mV. In vitro release studies at pH 7.4 demonstrated ultra-slow hydrolysis of amide bonds, with a CT release of 0.1–0.5% after 12 h; at pH 5.2, the hydrolysis rate slightly increased; however, it remained extremely low (1.5% of CT was released after 12 h). The antimicrobial activity of the conjugates depended on the DS. At DS 8%, the minimum inhibitory concentration (MIC) of the conjugate was equal to the MIC of native CT (1 µg/mL); at DS of 3 and 5%, the MIC increased 8-fold. In addition, the developed systems reduced CT nephrotoxicity by 20–60%; they also demonstrated the ability to reduce bacterial lipopolysaccharide-induced inflammation in vitro. Thus, these promising CT-SucCS conjugates are prospective for developing safe and effective nanoantibiotics.

## 1. Introduction

The continuous growth of microbial resistance to modern antimicrobial agents is a major challenge for today’s pharmacy and medicine and a significant threat to global health [1,2,3]. According to the World Health Organization (WHO) [3], there are common dangerous bacteria with drug resistance to penicillin (*Streptococcus pneumoniae*), fluoroquinolones (*Escherichia coli*, *Salmonella*, and *Shigella* species), as well as carbapenems and 3rd generation cephalosporins (*E. coli*, *Klebsiella pneumoniae*, and *Neisseria gonorrhoeae*). In 2017, the WHO published a list of antibiotic-resistant priority pathogens (e.g., *Acinetobacter*, *Pseudomonas*, and various *Enterobacteriaceae*) that often cause nosocomial pneumonia, posing the most risk to human health [4,5]. The peptide antibiotic colistin (CT) is effective against Gram-negative bacteria [6,7]; since the mechanism of action of CT is not associated with any specific receptors on the surface of the bacterial cell, the resistance of bacteria to CT is extremely rare [8,9]. Therefore, CT is a last-line antibiotic against severe nosocomial infections with multidrug resistance [10,11]. Microbial resistance to CT associated with MCR-like enzymes (MCR-1) has recently increased. Different plasmids can mediate the rapid transfer of MCR-1 between different bacterial species, and this is a crucial factor in the development of global microbial polyresistance to colistin [12]. The CT treatment of drug-resistant bacterial infections needs to use a higher drug dosage with higher toxicity and longer periods of hospitalization, resulting in decreased quality of life and increased mortality [13,14,15].

An attractive strategy to improve the therapeutic properties of antimicrobial agents (increased local bioavailability and reduced drug dose and systemic toxicity) is to develop nanosized drug delivery systems in the form of drug-polymer conjugates (so-called nanoantibiotics) [16,17,18]. This conjugation strategy can promote the following:

(i) multiple mechanisms of action against bacteria due to synergism and potentiation of the antimicrobial effect [19,20];
(ii) self-assembly of the resulting polymer systems into nanosize-structures for targeted antibiotic delivery in the infection inflammation site through the enhanced permeability and retention (EPR) effect [21], including intracellular targeting (as a result, the accumulation of higher doses at the site of infection at a lower total drug dose improves the biodistribution, maximizes the local bioavailability, and minimizes the systemic toxicity of the antimicrobial agent [16,22,23], as well as reduces the risk of side effects and the possibility of developing microbial resistance [2,24]);
(iii) controlled release and prolonged drug residence time in circulation;
(iv) the possibility of including both hydrophilic and hydrophobic components (expands the possibilities of pharmaceutical development of new antimicrobial formulations [25]);
(v) protection against enzymatic degradation and increased stability of the drug [19,20,26,27,28].

Natural polymers such as chitosan (CS), dextrin, gellan gum, and hyaluronic acid are widely used as drug carriers across the bacterial membrane; they are nearly ideal candidates for antibiotic conjugation due to their non-toxicity, biocompatibility, and biodegradability [18,29,30,31]. Moreover, CS is an appealing biopolymer for developing novel antibiotic delivery systems due to its ability to enhance the penetration of macromolecules through cell membranes. CS also provides sustained and targeted effects, which is a crucial requirement for an effective drug delivery system to the lungs [32,33].

In addition, various CS derivatives (e.g., succinyl chitosan (SucCS)) are used to prolong drug release due to slower biodegradation in the body compared to the native CS [34,35,36].

The main role of polymer in the conjugates is the targeted antibiotic delivery to the disease site, the improvement of biocompatibility, and the protection of the antibiotic from degradation by proteases and efflux channels [20,37]. As a result, the toxicity of the antimicrobial agents is reduced, with the potential for improvement in the antimicrobial activity.

When an antibiotic is covalently coupled to a polymer, various further mechanisms of conjugate action can be predicted. First, the antimicrobial activity may depend on the kinetics of antibiotic release, which is determined by the rate of hydrolysis (including enzymatic hydrolysis) of the corresponding chemical bond. Second, at high degrees of substitution (DS), the conjugated antibiotic can exhibit antimicrobial activity at the level of the original antibiotic. Such a case is possible if the active centers of the molecule responsible for the pharmacological effect are vacant and sterically available [18,38].

The aim of this study is to develop nanomedicine-based polymeric delivery systems for targeted delivery of CT for potential application as a nanoantibiotic against multidrug-resistant Gram-negative bacteria. CT acts as an inhibitor of cell membrane function [39], which makes it highly effective against microbial resistance. However, native CT has high nephro- and neurotoxicity [40,41]. We hypothesize that conjugation of CT with CS previously modified via succinic anhydride provides the formation of negatively charged nanoparticles suitable for intravenous administration. This strategy can improve the targeted delivery of CT in bacterial inflammation sites by the EPR effect as well as cellular permeability (e.g., macrophages) with subsequent prolonged drug release and decrease of toxicity while maintaining pharmacological activity.

## 2. Results

### 2.1. Synthesis and Characterization of Succinyl Chitosan

To introduce a carboxyl group, CS was modified with a succinyl linker. SucCS was synthesized using a 5-fold molar excess of SA for 5 h in an acetic acid medium under homogeneous conditions [42]. The resulting polymer was characterized by ^1^H NMR spectroscopy (400 MHz, D_2_O/TFA) δ 2.08 (s, J = 0.78 Hz Ac); 2.70 (d, J = 2.80 Hz CH_2_); 3.21 (t, J = 0.32 Hz, H–2 GluNH_2_); 3.5–4.0 (m, J = 5.95 Hz, H–3,4,5,6 H–2 GluNHAc), 4.62 (d, J = 0.68 Hz, H–1, H–2 GluNH_2_, GluNHAc); 4.94 (d, J = 0.34 Hz, H–1 GluNH_2_) [42]. The DS, according to ^1^H NMR spectroscopy data, was 70%. The high DS of SucCS is necessary to form a negative surface charge of the polymeric particles after CT grafting to ensure their compatibility with blood components [43].

The resulting CS derivative with a succinyl linker was used for conjugation with CT.

### 2.2. Synthesis and Characterization of Succinyl Chitosan-Colistin Conjugates

**Figure 1:**
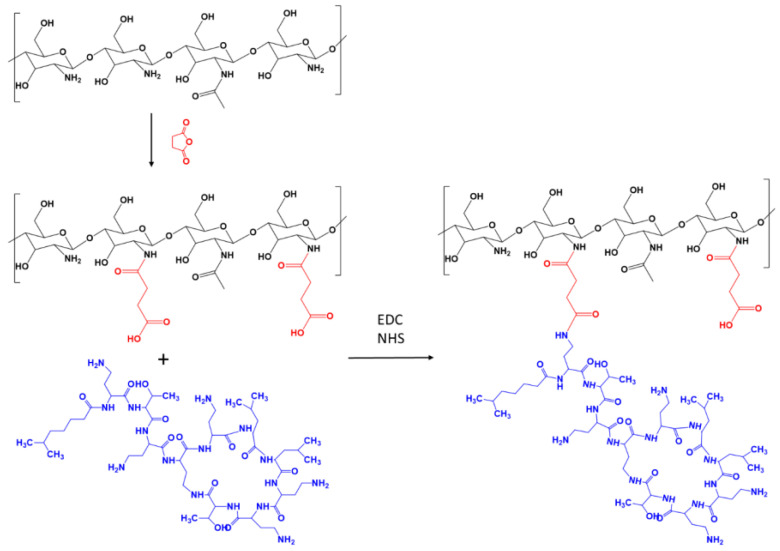
Synthesis scheme for succinyl chitosan-colistin conjugate (SucCS-CT).

In the present study, SucCS-CT was synthesized in an aqueous solution via the EDC-mediated coupling between amine groups of CT and carboxyl groups of SucCS in the presence of NHS (Figure 1). The pH of the reaction mixture was about 5 and did not change significantly during the reaction process. Dialysis against 0.9% NaCl solution was used to remove non-conjugated CT from the final reaction mixture.

**Figure 2:**
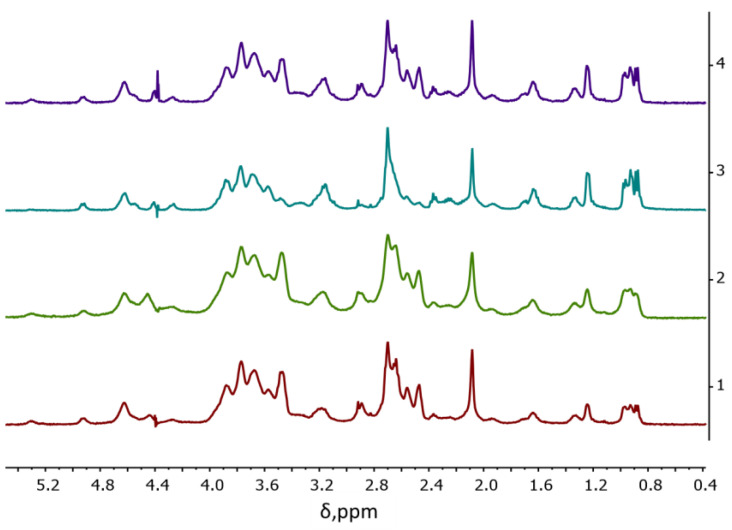
Stacked 1H NMR spectra of succinyl chitosan-colistin conjugates (SucCS-CT) samples: 1-SucCS-CT-3; 2-SucCS-CT-5; 3-SucCS-CT-10; 4-SucCS-CT-15.

The DSs of SucCS-CT (Table 1) were calculated from the ^1^H NMR spectra (Figure 2) using the CT multiplet signal at 0.74–0.88 ppm (*I*_0.74–0.88_) corresponding to 18 CT protons (Figures S1 and S2) by the following equation $DS_{CT}=\frac{I_{0.74–0.88}}{18I\left(H-1\right)}$. The signal of the methyl protons of the SucCS acetamide group at 2.08 ppm was taken as a reference signal (0.78 H for CS with a degree of acetylation of 26%).

The reaction of CT with SucCS at various molar ratios (0.03, 0.05, 0.1, and 0.15) leads to the formation of water-soluble conjugates with different degrees of substitution (DS were 3, 5, and 8%, respectively). The EC was about 100% at SucCS:CT molar ratios of 1:0.03 and 1:0.05 and 81% at a molar ratio of 1:0.10 (Table 1). However, when we used a SucCS:CT molar ratio of 1:0.15, the EC reached only 54%. Therefore, increasing the CT content in the reaction mixture above 10 mol% relative to SucCS is impractical. The CT content was 130, 187, and 311 μg/mg for the syntheses with SucCS:CT molar ratios of 1:0.03, 1:0.05, and 1:0.10, respectively (Table 1).

**Figure 3:**
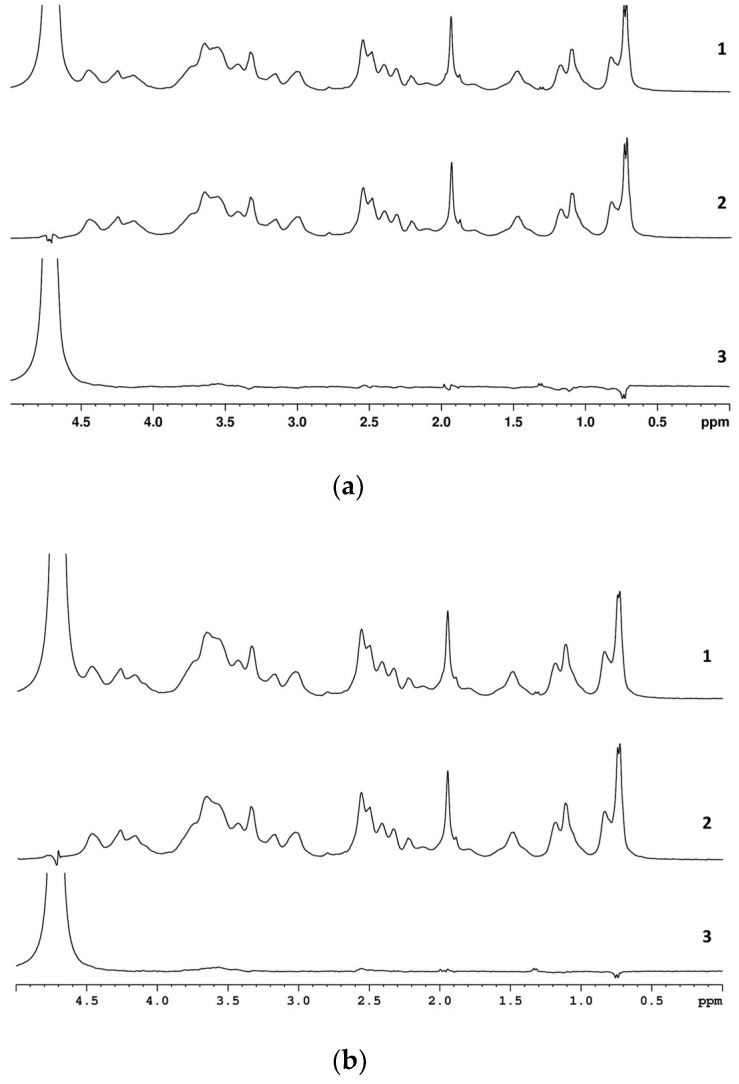
1D-DOSY spectra of succinyl chitosan-colistin conjugate (SucCS-CT-10) in D_2_O/TFA (**a**) and in PBS/D_2_O/TFA (**b**): g = 5% (1); g = 90% (2); difference spectrum (3).

To prove the covalent conjugation of CT to SucCS, we used the 1D-DOSY spectroscopy (Figure 3). We have previously shown [7] that CT is able to form stable polyelectrolyte complexes with anionic polymers in water; however, these complexes are dissociated in a PBS solution. Therefore, we used both D_2_O and PBS in D_2_O as NMR solvents to eliminate the possible influence of ionic interaction on the diffusion coefficient.

The 1D-DOSY experiments showed that the signal intensities of CT and SucCS varied by the same factor when changing the amplitude of the magnetic field gradient (5% and 90% of the maximum value). Thus, CT was conjugated to SucCS via covalent bonding, and both experienced the same translational diffusion despite the significant difference in the molecular sizes of SucCS and CT.

### 2.3. Physicochemical Characterization of the Succinyl Chitosan-Colistin Conjugates

The developed amphiphilic conjugates self-assembled in aqueous media into negatively charged nanostructures with a size of about 100–200 nm (Table 2).

The size of the formed particles depended on the DS; with an increase in the DS from 3 to 8%, the particle size increased almost 2-fold (on a mean from 100 to 200 nm). The conjugates were also characterized by NTA (Figure S3); the mean particle sizes measured by DSL and NTA correlated with each other (Table 2). The size of polymeric particles is a crucial factor for their successful interaction with the biological environment after administration (vascular distribution and tissue permeability, molecular binding to cells, and intracellular transport), thereby determining the total delivery efficiency. It is known that particles between 10 and 200 nm are most suitable for physical and biochemical targeting for intravascular and site-specific delivery [44].

The resulting conjugates had a negative surface charge (ζ-potential), which also depended on the number of grafted CT moieties. With an increase in DS from 3 to 8%, the ζ-potential decreased from −28 to −22 mV, which is obviously due to the increase in the presence of protonated positively charged CT amino groups.

**Figure 4:**
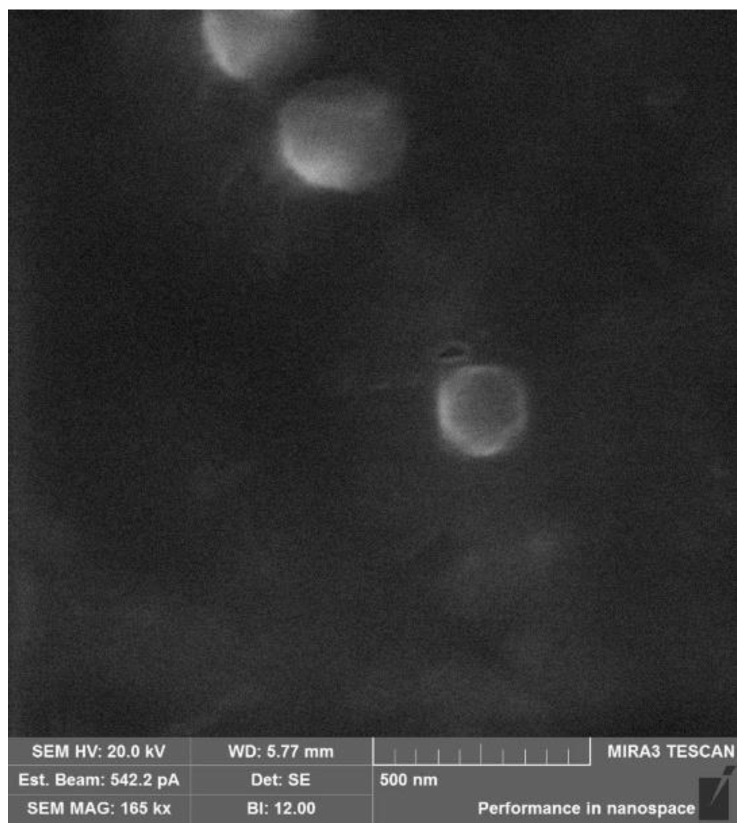
SEM images of the succinyl chitosan-colistin conjugates (SucCS-CT-10).

The SEM image showed spherically symmetric nanoparticles with a diameter of about 200 nm, which does not conflict with the light scattering data. (Figure 4).

### 2.4. Colistinrelease Kinetics from the Succinyl Chitosan-Colistin Conjugates

**Figure 5:**
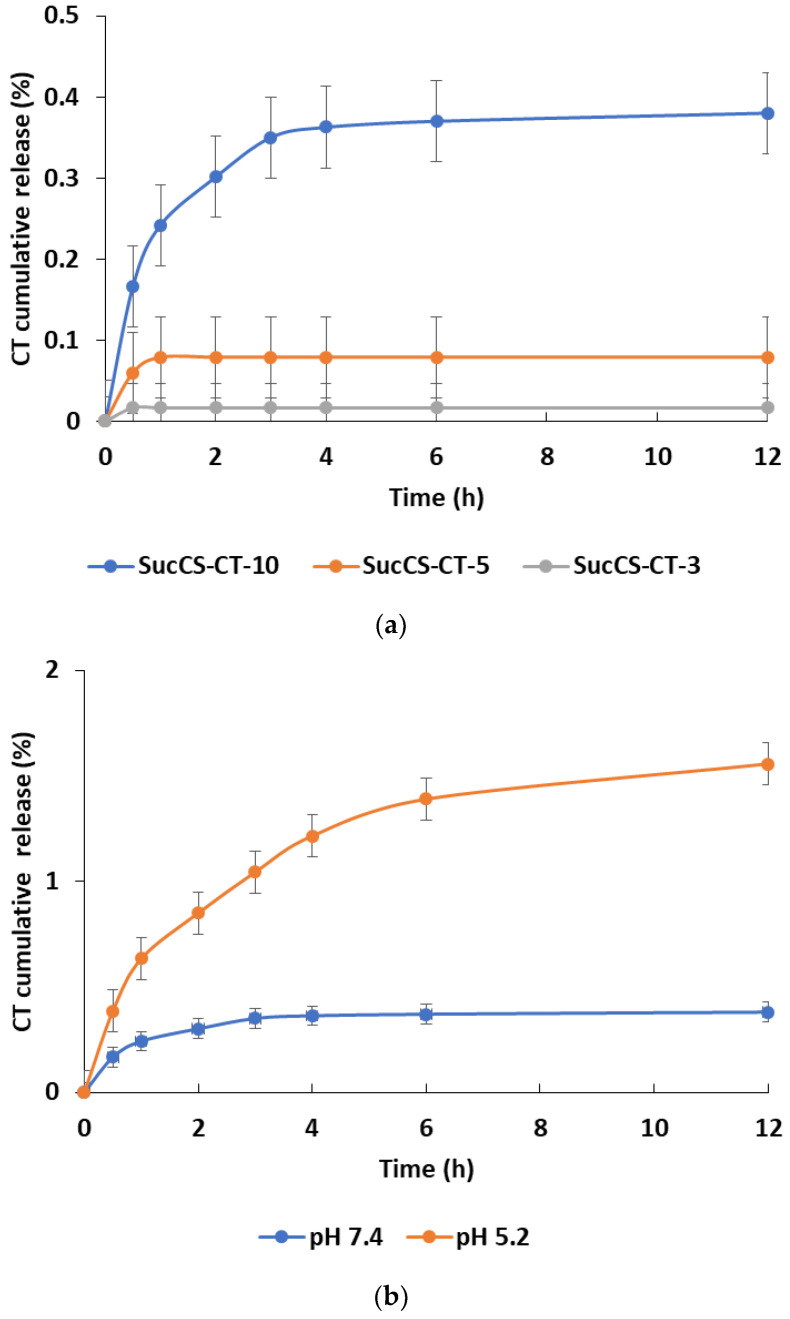
Colistin (CT) release kinetics at 37 °C from the succinyl chitosan-colistin conjugates (SucCS-CT-3, SucCS-CT-5, SucCS-CT-10) in PBS, pH 7.4 (**a**) and from SucCS-CT-10 in phosphate buffer, pH 5.2 (**b**). Each point represents the average of triplicate measurements ± SD.

Since the synthesized conjugates are supposed to be used for a parenteral application, the CT release was primarily studied in PBS (pH 7.4), simulating physiological conditions in the bloodstream. It was shown that CT was practically not hydrolyzed under these conditions within 24 h; the cumulative CT release was 0–0.1% for conjugates with DS of 3 and 5%, and 0.4% for conjugates with DS of 8%, respectively (Figure 5a). However, the environment of the bacterial inflammation sites has a locally acidic pH, and therefore it is important to study the CT release kinetics from the obtained conjugates under these conditions (phosphate buffer, pH 5.2). An in vitro study of the CT release profile from SucCS-CT-10 showed that within 24 h, CT was not released in both PBS (pH 7.4) and phosphate buffer (pH 5.2); the cumulative CT release under these conditions was 0.4% and 1.5%, respectively (Figure 5b).

### 2.5. Antimicrobial Activity of the Succinyl Chitosan-Colistin Conjugates

**Figure 6:**
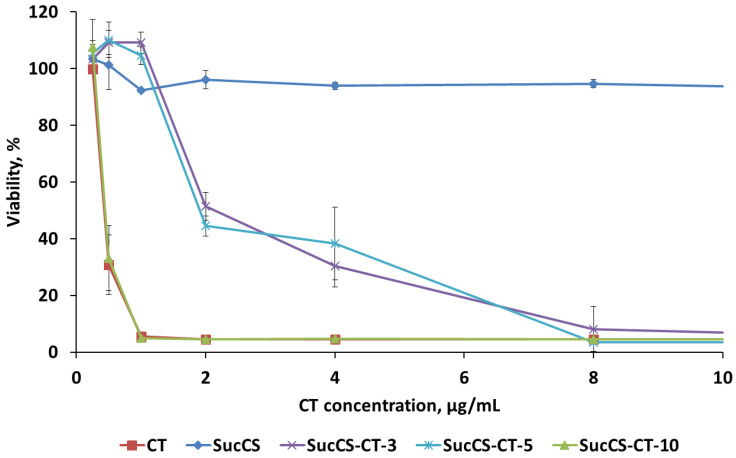
Antibacterial activity of succinyl chitosan-colistin conjugates against *P. aeruginosa*. Each point is represented as the mean of measurements ± SD.

The antibacterial activity of SucCS-CT samples was evaluated by minimal inhibitory concentration (MIC) for *P. aeruginosa* at the concentration of 1 × 10^7^ CFU/mL (Figure 6). The SucCS sample was also tested as a control. As seen in Figure 5, the SucCS sample itself does not significantly reduce the viability of the bacterial culture at high concentrations. In contrast, the SucCS-CT samples demonstrated antimicrobial effects. SucCS-CT-10 demonstrated antibacterial activity comparable to those of native CT. The MICs of SucCS-CT-10 and native CT were 1 μg/mL, while MICs of other samples, such as SucCS-CT-3 and SucCS-CT-5, against *P. aeruginosa*, were 8 μg/mL. It should be noted that SucCS-CT-3 and SucCS-CT-5 at the concentrations of 0.25–1 μg/mL increased the viability of *P. aeruginosa* by 5–10%.

### 2.6. Cytotoxicity of the Succinyl Chitosan-Colistin Conjugates

**Figure 7:**
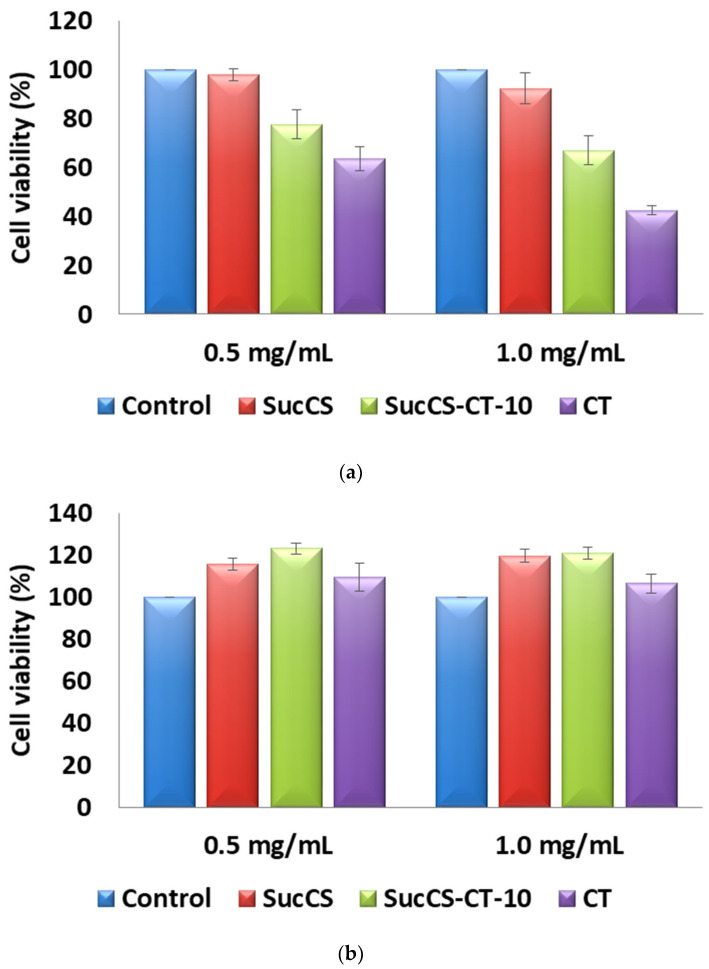
Viability of HEK 293 (**a**) and T 98G (**b**) cells incubated in the presence of SucCS-CT-10, SucCS, and native CT for 72 h. The CT concentrations in the tested samples were 0.5 and 1.0 mg/mL. Data are presented as mean ± SD (*n* = 5).

Based on the high antimicrobial activity, SucCS-CT-10 was chosen as a promising polymeric CT delivery system for cytotoxicity studies. We examined the potential nephron- and neurotoxicity of the resulting SucCS-CT conjugates and, as control, the equivalent amounts of CT and SucCS on renal (HEK 293) and brain (T 98G) cell lines. In vitro cytotoxicity experiments showed that the SucCS-CT conjugate increased cell viability compared with native CT 1.2 and 1.6-fold for CT concentrations of 0.5 and 1.0 mg/mL, respectively (Figure 7a). The studied CT concentrations had no toxic effect on glioblastoma cells; however, even in this experiment, the cell viability in the presence of conjugated CT was about 13% higher than in the presence of native CT (Figure 7b). Therefore, the SucCS-CT conjugates indeed reduced the toxicity of CT.

### 2.7. Anti-Inflammatory Activity of the SucCS-CT Conjugates

Primarily, we investigated THP-1 cell viability in response to SucCS-CT-10 conjugate stimulation. We found that CT in final concentrations of 1000 and 500 µg/mL and SucCS-CT-10 in final concentrations of 1000 µg/mL significantly (*p* < 0.001 in all three cases) decreased the frequencies of alive THP-1 cells after 24 h in vitro incubation (Table 3).

Next, we investigated the effects of SucCS-CT-10 conjugate stimulation on CD54 expression by THP-1 cells and found that SucCS, native CT, and SucCS-CT-10 in final concentrations of 1000 and 500 µg/mL increased CD54 expression of cell membranes of THP-1 cells. Furthermore, SucCS-CT-10 conjugate in a final concentration of 100 µg/mL significantly activated THP-1 cells (Table 4).

Finally, we tested the anti-inflammatory activity of SucCS-CT-10 conjugate in LPS-treated THP-1 cells (Table 5). Primarily, we noticed that in vitro stimulation of THP-1 cells with bacterial LPS and SucCS-CT-10 conjugate simultaneously had no effect on cell viability (Table S1). Next, we found that SucCS had no influence on LPS activation over the tested range of concentrations. Furthermore, native CT effectively down-regulated CD54 expression on LPS-treated THP-1 cells at all concentrations starting from 500 µg/mL. Similarly, SucCS-CT-10 conjugate significantly inhibited LPS-induced CD54 expression of THP-1 cells (Table 5).

## 3. Discussion

Pharmaceutical development of new substances with antimicrobial activity is a long-term, high-cost, and complex regulatory process [45]. Therefore, the modification of known antimicrobial agents or screening of such candidates among compounds approved for medical use represents a promising trend [46,47]. For example, our research group [18] studied the grafting of CT to hyaluronic acid via amide bond formation. The resulting compound had different DS (3–8%), and they were chemically stable (CT release was 1–5% after 24 h). The conjugates with high DS (8%) retained antimicrobial activity at the level observed with native CT (1 µg/mL). At the same time, the toxicity of conjugated CT was reduced by 1.5-fold. Ferguson et al. [30,38] synthesized a dextrin-CT conjugate. The conjugates based on low molecular weight (MW) succinylated dextrin (MW = 7500, DS = 0.01) demonstrated the optimal release profile of CT in the presence of physiological amylase concentrations: 80% of the drug was released from the developed conjugates within 48 h. From the reference drug (prodrug CT sodium methanesulfonate), 33% of CT was released during the same time. These conjugates exhibited antimicrobial activity comparable to native CT in in vitro experiments against gram-negative pathogens. However, in vitro toxicity to kidney cells was reduced 13-fold. In vivo experiments in rats demonstrated improved pharmacokinetics of the conjugates, prolonged the antimicrobial effect 12-fold, and decreased in vivo toxicity. Peng et al. [31] conjugated polymyxin B with deacylated gellan gum; obtained polymeric systems were characterized by the high conjugating rate (96%) and the sustained release (30% of C within 60 h). The conjugates effectively inhibit liposaccharide-stimulated cytotoxic nitric oxide overexpression by BV-2 cells, and the half-maximal inhibitory concentration value against BV-2 cells has increased by almost 130-fold compared to native polymyxin B.

Thus, the chemical conjugation of antimicrobial compounds with polymers is indeed a reliable way to produce targeted CT delivery systems with programmable and controlled release as well as reduced cytotoxicity.

## 4. Materials and Methods

### 4.1. Materials and Reagents

CS from crab shell (Bioprogress, Shchelkovo, Russia) with an average viscosity MW (Mη) of 37,000 and an acetylation degree (DA) of 26% was used [48]. The intrinsic viscosity [η] of the CS was determined by capillary viscometry using an Ubbelohde viscometer (Design Bureau Pushchino, Russia) at 20 °C with 0.33 M acetic acid/0.3 M NaCl as solvent. The Mη of CS was calculated using the Mark-Houwink equation [η] = 3.41 × 10^−3^ × Mη^1.02^ [49]; [η] = 1.56 dL/g.

CT sulfate (MW of 1390) was obtained from BetaPharm (Wujiang, Shanghai, China). The contents of CT A (polymyxin E1) and CT B (polymyxin E2) were 31.1 ± 0.4% and 68.9 ± 0.4%, respectively, as determined previously by liquid chromatography-mass spectrometry (LC-MS) [50].

Phosphate buffered saline (PBS), Na_2_HPO_4_, KH_2_PO_4_, NaCl, NaHCO_3_, succinic anhydride (SA), 1-ethyl-3-(3-dimethylaminopropyl) carbodiimide hydrochloride (EDC), N-hydroxysuccinimide (NHS), acetic acid, trifluoroacetic acid (TFA), and D_2_O (99.9 atom % D) were obtained from Sigma-Aldrich Co. (St. Louis, MO, USA). All other reagents and solvents were of analytical grade and used as received.

### 4.2. Synthesis of the Succinyl Chitosan

Succinyl chitosan (SucCS) was prepared as described previously [42]: CS (0.1 g, 0.53 mmol of N) was dissolved in 1% acetic acid solution (5 mL), and then a 5-fold molar excess of succinic anhydride was added. The reaction mixture was stirred for 5 h at room temperature. The resulting product was precipitated with acetone, dissolved in a 3% NaHCO_3_ solution, dialyzed against deionized water for three days, and then freeze-dried. The finished product was characterized using the NMR method (see Section 4.4) and elemental analysis. The elemental analysis was performed on a Vario EL CHN analyzer (Elementar, Langenselbold, Germany).

### 4.3. Synthesis of the Succinyl Chitosan-Colistin Conjugates

Succinyl chitosan-colistin conjugates (SucCS-CT) were prepared by the following method [18]: SucCS (0.1 g, 0.36 mmol of N) was dissolved in distilled water (20 mL), and then 2-fold molar quantities of EDC and NHS were added for activation of the SucCS carboxyl groups. The reaction mixture was stirred for 0.5 h at 40 °C. Then a 1 mL CT solution was added (see Table 1 for CT quantities), and the resulting reaction mixture was stirred overnight at 40 °C. The final product was dialyzed against 0.9% NaCl solution for three days, then distilled water for two days, and finally freeze-dried.

### 4.4. NMR Characterization of the Succinyl Chitosan and Succinyl Chitosan-Colistin Conjugates

Samples (5 mg) of SucCS or SucCS-CT were dissolved in D_2_O/TFA or PBS/D_2_O/TFA. The ^1^H NMR spectra were recorded using Bruker Avance 400 (Bruker, Billerica, MA, USA) spectrometer at 70 °C using a zgpr pulse sequence with residual H_2_O suppression. The one-dimensional diffusion-ordered (1D-DOSY) NMR spectra were recorded at 24 °C using a stebpgplsld pulse sequence. The amplitude values of the magnetic field gradients (g) were 5% and 90% of their maximum value. The diffusion time was 90 ms, and the gradient duration was 2 ms.

### 4.5. Determination of Colistin Content in the Succinyl Chitosan-Colistin Conjugates and Conjugation Efficiency

The CT content in the conjugates and conjugation efficiency (CE) were determined using an indirect method by measuring the concentration of the non-conjugated CT. The reaction mixture (see Section 4.3) was separated by ultrafiltration at 4500 rpm through a 10,000 MWCO Vivaspin^®^ Turbo 4 centrifugal concentrator, and then the precipitate was washed twice with 4 mL of 0.9% NaCl solution to remove the ion-bound CT. CT concentration in the collected solution was analyzed by LC-MS according to the protocol described in [18]. The CT content and CE were calculated using the following equations:

(1) $$\mathrm{CT}\;\mathrm{content}\;(\mu g/\mathrm{mg})=\frac{\mathrm{Total}\;\mathrm{CT}\;\mathrm{mass}\;-\;\mathrm{CT}\;\mathrm{mass}\;\mathrm{in}\;\mathrm{supernatant}\;\left(\mu g\right)}{\mathrm{Total}\;\mathrm{SucCS}\;\mathrm{mass}+\left(\mathrm{Total}\;\mathrm{CT}\;\mathrm{mass}\;-\mathrm{CT}\;\mathrm{mass}\;\mathrm{in}\;\mathrm{supernatant}\right)\left(\mathrm{mg}\right)}$$

(2) $$\mathrm{CE}\;(\%)=\frac{\mathrm{Total}\;\mathrm{CT}\;\mathrm{mass}\;-\;\mathrm{CT}\;\mathrm{mass}\;\mathrm{in}\;\mathrm{supernatant}\;\left(\mu g\right)}{\mathrm{Total}\;\mathrm{CT}\;\mathrm{mass}\;\left(\mu g\right)}$$

### 4.6. Physicochemical Characterization of the Succinyl Chitosan-Colistin Conjugates

The mean size of the formed nanoparticles based on SucCS-CT was measured by dynamic light scattering (DLS) using Photocor Compact-Z instrument (Photocor, Moscow, Russia), as well as by nanoparticle tracking analysis (NTA) using Nanosight NS300 (Malvern Panalytical Ltd, Malvern, UK). The DLS measurements of SucCS-CT (0.5 mg/mL) were performed in PBS medium (pH 7.4) at 20 °C with a fixed scattering angle of 90°.

The NTA software tracks multiple particles under Brownian motion and relates the velocity of particle movement with their size, and then calculates their hydrodynamic diameters using the Stokes-Einstein equation [51,52]. For NTA measurements, SucCS-CT solutions (0.1 mg/mL) in PBS (pH 7.4) were diluted 250-fold with ultrapure distilled water, and results were expressed in particle size (nm) and particle size distribution.

The ζ-potential of obtained polymeric systems in PBS solution (pH 7.4) was measured by the method of electrophoretic DLS using the ZetasizerNano-ZS (Malvern Panalytical Ltd, Malvern, UK).

All analyses were performed in triplicate (*n* = 3), and the data were expressed as the mean values and standard deviations.

The morphology of the SucCS-CT was imaged by scanning electron microscopy (SEM) using a Tescan Mira 3 instrument (Tescan, Brno, Czech Republic). SEM images were obtained in the secondary electron mode with an accelerating voltage of 20 kV. Prior to SEM measurements, the samples were placed on double-sided carbon tape and dried in vacuo for 48 h.

### 4.7. In Vitro Colistin Release from the Succinyl Chitosan-Colistin Conjugates

The release medium conditions were chosen based on the FDA recommendations for dissolution methods for modified injectable dosage forms and have been previously reported [7]. A SucCS-CT sample (1 mg) was dissolved in PBS (1 mL, pH 7.4) or phosphate buffer (1 mL, pH 5.2) and incubated at 37 °C. At regular intervals, 1 mL of medium was ultracentrifuged at 4500 rpm using a 10,000 MWCO Vivaspin^®^ Turbo 4 centrifugal concentrator. The mass of the released CT was determined in the supernatant by LC-MS as previously reported [18].

### 4.8. Antimicrobial Activity of the Succinyl Chitosan-Colistin Conjugates

The antimicrobial activity was tested using the microtiter broth dilution method and a *P. aeruginosa* ATCC 27853 culture (Museum of Microbiological Cultures, State Research Institute of Highly Pure Biopreparations, St. Petersburg, Russia) as previously described [18]. Stock solutions of SucCS-CT were prepared by diluting the samples in Mueller-Hinton broth to a maximum 2-fold required CT concentration. Then the serial dilutions of CT at concentrations from 64 to 0.25 μg/mL were prepared.

The optical density (OD) of an overnight P. aeruginosa suspension in Mueller-Hinton broth was measured with a UV mini-1240 spectrophotometer (Shimadzu, Kyoto, Japan) at a wavelength of 540 nm. The culture suspension was serially diluted 1:100 in Mueller-Hinton broth to give approximately 1 × 10^7^ CFU/mL. The number of colony-forming units was controlled by parallel plated of the culture suspension on Luria-Bertani agar.

The inoculum was prepared by cultivation in a liquid medium (Mueller-Hinton broth) with SucCS-CT solutions by adding 125 μL of inoculum to the wells of the culture plate. The plate also contained controls: 100% growth (bacteria only), sterility control (Mueller-Hinton broth only), and SucCS at equivalent polymer concentrations. The plate was incubated for 24 h at 37 °C; the OD was measured on an ELx808™Absorbance Microplate Reader (BioTek Instruments, Winooski, VT, USA) at 630 nm. Each sample was tested triplicate in three independent series (*n* = 9).

### 4.9. Cytotoxicity Study of the Succinyl Chitosan-Colistin Conjugates

Cytotoxicity tests were carried out according to the previously reported method [18] using a human kidney embryonic cell line (HEK 293) and a human glioblastoma cell line (T 98G). The cells were cultivated at 37 °C in a humidified 5% CO_2_ atmosphere in EMEM culture medium (Eagle’s minimal essential medium; Gibco, Billings, MT, USA) containing 1% essential amino acids, 10% (*v*/*v*) thermal inactivated fetal bovine serum (FBS; HyClone Laboratories, Logan, UT, USA), 1% L-glutamine, 50 U/mL penicillin, and 50 μg/mL streptomycin. For the experiment, 5.0 × 10^3^ cells/100 μL/well were seeded into 96-well plates and cultured for 24 h, followed by 100 or 50 μL of the test substance solutions (containing 1 mg of CT and equivalent amounts of SucCS-CT-10 or SucCS) in EMEM medium with FBS for 24 h. The medium was removed, and test compounds were added to the cells. For this, 50 μL of pure culture medium was added to part of the wells to obtain sample concentrations equivalent to 0.5 mg of CT (to study the dose-dependent effect), and the cells were incubated for 72 h. At the end of the incubation period, the medium was removed, and 50 μL of EMEM medium containing 0.1 mg/mL 3-(4,5-dimethylthiazol-2-yl)-2,5-diphenyl-tetrazolium bromide (MTT reagent) was added to each well. The cells were incubated in a CO_2_ incubator for 2 h at 37 °C. After removal of the supernatant, the formazan crystals formed by metabolically viable cells were dissolved in dimethyl sulfoxide (50 μL/well), and the optical density was measured at 570 nm with a UV mini-1240 spectrophotometer (Shimadzu, Kyoto, Japan). Calculations were performed using polynomial regression analysis in Microsoft Excel.

### 4.10. Anti-Inflammatory Activity of the SucCS-CT Conjugates

Human monocytic leukemia cells (THP-1 cells) were used as model objects for the experiment with SucCS-CT blockade of LPS-induced activation. THP-1 cells were maintained in RPMI 1640 medium (Biolot, St. Petersburg, Russia) supplemented with 10% heat-inactivated dialyzed fetal calf serum (FBS, Gibco), 50 μg/mL gentamicin (Biolot, St. Petersburg, Russia), and 2 mM L-glutamine (Biolot, St. Petersburg, Russia) as it was described in details previously [53]. Primarily, we investigated cell viability using PO-PRO-1 (final concentration 250 nM; Molecular Probes, Eugene, OR, USA) and DRAQ7 (final concentration 3 μM, Beckman Coulter, Indianapolis, IN, USA) staining as described previously [54]. Next, THP-1 cells were activated in vitro by adding 2 ng/mL of lipopolysaccharides (LPS) from *Escherichia coli* (Sigma-Aldrich, Merck KGaA, Darmstadt, Germany), while untreated cells were used as a negative control. The test compounds (SucCS, CT, and SucCS-CT) were added to the wells at final concentrations of 1000 and 1 µg/mL and incubated with THP-1 cells for 24 h. Finally, the cells were transferred to flow cytometry tubes and were stained with mouse anti-human CD54-PE antibodies (Beckman Coulter Inc., Indianapolis, IN, USA), as described previously [35]. No less than 10,000 single THP-1 cells were analyzed per sample. Flow cytometry data were obtained with a Navios™ flow cytometer (Beckman Coulter, Beckman Coulter Inc., Indianapolis, IN, USA) equipped with 405, 488, and 638 nm lasers and analyzed using Navios Software v.1.2 and Kaluza™ software v.2.0 (Beckman Coulter, Beckman Coulter Inc., Indianapolis, IN, USA). The data were presented as the percentage of viable cells per sample ± standard error (SE), and the intensity of CD54 expression was ultimately measured as mean fluorescence intensity (MFI) on the cell surface of viable THP-1 cells.

## 5. Conclusions

This work had several key aspects:

(i) A two-step synthetic procedure was developed for the carbodiimide conjugation of the peptide antibiotic CT with CS via a succinyl linker to form a slow hydrolyzable amide bond. For this purpose, the initial CS was succinylated previously to achieve a DS of 70%. Then, using different molar ratios of the reactants, SucCS-CT with different DS (ranging from 3 to 8%) was obtained; the CT content was 130–318 μg/mg. It was demonstrated that the CE decreased from 80–100% to 50% when the CT in the reaction mixture was increased from 3–10 to 15 mol%. Thus, increasing the amount of CT above 10 mol% relative to SucCS is not reasonable.
(ii) The resulting systems formed associates in an aqueous solution (PBS, pH 7.4) with a ζ-potential of −22 to −28 mV and a hydrodynamic diameter of 100–200 nm that provided both good physical colloidal stability and aggregative resistance against blood components due to suitable surface charge as well as improved delivery through improved vascular distribution, tissue permeability, molecular binding, and intracellular transport due to physiological size.
(iii) In vitro studies demonstrated that the developed conjugates practically did not release CT for 12 h under both physiological and inflammation site conditions (about 0.4–1.5% for 12 h). The body environment has its own characteristics related to the presence of enzymes that can affect the hydrolysis and release of the drug from the drug-polymer conjugates. However, succinylated CS is less biodegradable than the original CS; therefore, SucCS is an excellent polymer carrier for sustained-release delivery systems.
(iv) At the same time, the antimicrobial activity of the obtained conjugates depended on the DS; the SucCS-CT with high DS (about 8%) exhibited comparable antimicrobial activity with native CT (1 μg/mL). In this case, the rate of conjugate hydrolysis is not a determinant of its pharmacological efficacy.
(v) Nevertheless, conjugation of CT with SucCS reduced nephrotoxicity (HEK 293 cells) compared with native CT (20–60% lower) and neurotoxicity (T 98G cells). Since the decrease in cytotoxicity is related to the covalent grafting of CT to the polymer, slow hydrolysis, in this case, has an important positive effect.
(vi) Finally, the obtained conjugates exhibited anti-inflammatory activity like native CT in an LPS-induced inflammation model in vitro; however, the conjugation of CT with SucCS reduced the cytotoxic activity of CT against THP-1 cells. Moreover, unlike native CT, SucCS-CT conjugates at low concentrations (10–1 μg/mL) exhibited low cytotoxic and high anti-inflammatory activity, which may be useful for developing a new anti-inflammatory agent.

We suppose that this study can be continued in in vivo biological tests to develop a new generation of effective nanoantibiotics to combat multidrug resistance of Gram-negative pathogens.

## Figures and Tables

**Table 1:** Condition for synthesis and characterization of succinyl chitosan-colistin conjugate (SucCS-CT).

Sample	Molar Ratio of the Reactants				DS (%)	CE (%)	CT-Content (μg/mg)
	SucCS	CT	EDC	NHS			
SucCS-CT-3	1	0.03	2	2	3	100.0 ± 0.8	130 ± 1
SucCS-CT-5	1	0.05	2	2	5	99.8 ± 1.1	187 ± 2
SucCS-CT-10	1	0.10	2	2	8	81.0 ± 1.6	311 ± 5
SucCS-CT-15	1	0.15	2	2	8	54.4 ± 1.5	318 ± 5

**Table 2:** Physico-chemical properties of the succinyl chitosan-colistin conjugates.

Sample	Hydrodynamic Diameter ± SD (nm)		ζ-Potential ± SD (mV)
	by DSL	by NTA	
SucCS-CT-3	114 ± 17	113 ± 58	−27.7 ± 0.5
SucCS-CT-5	149 ± 16	137 ± 40	−26.0 ± 0.9
SucCS-CT-10	180 ± 21	202 ± 67	−22.4 ± 0.6

**Table 3:** Viability of THP-1 cells after 24 h in vitro co-cultivation with SucCS-CT conjugate. Data are presented as mean ± SD (*n* ≥ 6); the data are shown as the percentages of viable cells.

No Stimulation 94.5 ± 0.2			
Concentration	SucCS	CT	SucCS-CT-10
1000 µg/mL	94.3 ± 0.4	69 ± 2 ***	85 ± 3 ***
500 µg/mL	94.7 ± 0.4	84.9 ± 0.6 ***	93.7 ± 1.0
100 µg/mL	93.7 ± 0.4	93.3 ± 0.5	92.9 ± 0.6
10 µg/mL	94.9 ± 0.4	94.7 ± 0.5	94.4 ± 0.5
1 µg/mL	94.8 ± 0.5	94.2 ± 0.5	95.07 ± 0.18

***—the difference with negative control (THP-1 cell without stimulation) were significant with *p* < 0.001 according to non-parametrical Mann–Whitney U test.

**Table 4:** CD54 expression by THP-1 cells in response to in vitro stimulation with SucCS-CT-10 conjugate. Data are presented as mean ± SD (*n* ≥ 6); the data are shown as CD54 MFI.

No Stimulation 0.685 ± 0.017			
Concentration	SucCS	CT	SucCS-CT-10
1000 µg/mL	8.7 ± 1.4 ***	1.61 ± 0.08 ***	10.3 ± 1.8 ***
500 µg/mL	2.1 ± 0.2 ***	0.960 ± 0.061 ***	3.0 ± 0.4 ***
100 µg/mL	0.82 ± 0.06	0.720 ± 0.015	0.93 ± 0.06 ***
10 µg/mL	0.663 ± 0.016	0.674 ± 0.015	0.72 ± 0.03
1 µg/mL	0.664 ± 0.016	0.653 ± 0.017	0.69 ± 0.04

***—the difference with negative control (THP-1 cell without stimulation) were significant with *p* < 0.001 according to non-parametrical Mann–Whitney U test.

**Table 5:** Effects of SucCS-CT-10 conjugate on LPS-induced CD54 expression of THP-1 cells. Data are presented as mean ± SD (*n* ≥ 6); the data are shown as CD54 MFI.

LPS-Treated Cells 8.7 ± 1.3			
Concentration	SucCS	CT	SucCS-CT-10
1000 µg/mL	11 ± 2	2.11 ± 0.18	9.8 ± 1.9
500 µg/mL	4.6 ± 0.5	1.5 ± 0.3 **	2.3 ± 0.3 **
100 µg/mL	5.1 ± 0.6	0.85 ± 0.04 ***	1.03 ± 0.06 ***
10 µg/mL	8.8 ± 1.2	0.89 ± 0.07 ***	1.14 ± 0.16 ***
1 µg/mL	7.9 ± 1.1	0.99 ± 0.14 ***	1.47 ± 0.18 ***

** and ***—the difference with negative control (LPS-treated THP-1 cells) were significant with *p* < 0.01 and *p* < 0.001, respectively, according to non-parametrical Mann–Whitney U test.

## Data Availability

The data are contained within the article and Supplementary Materials.

## Supplementary Materials

The following supporting information can be downloaded at: https://www.mdpi.com/article/10.3390/ijms24010166/s1.
