# Supplementary material for: Succinyl Chitosan-Colistin Conjugates as Promising Drug Delivery Systems
Source: Int J Mol Sci. 2022 Dec 22;24(1):166. doi: 10.3390/ijms24010166 (PMC9820547; doi:10.3390/ijms24010166)
Supplement: Supplementary file 1 [file ijms-24-00166-s001.zip › ijms-2081258-supplementary.pdf]

Supplementary Materials

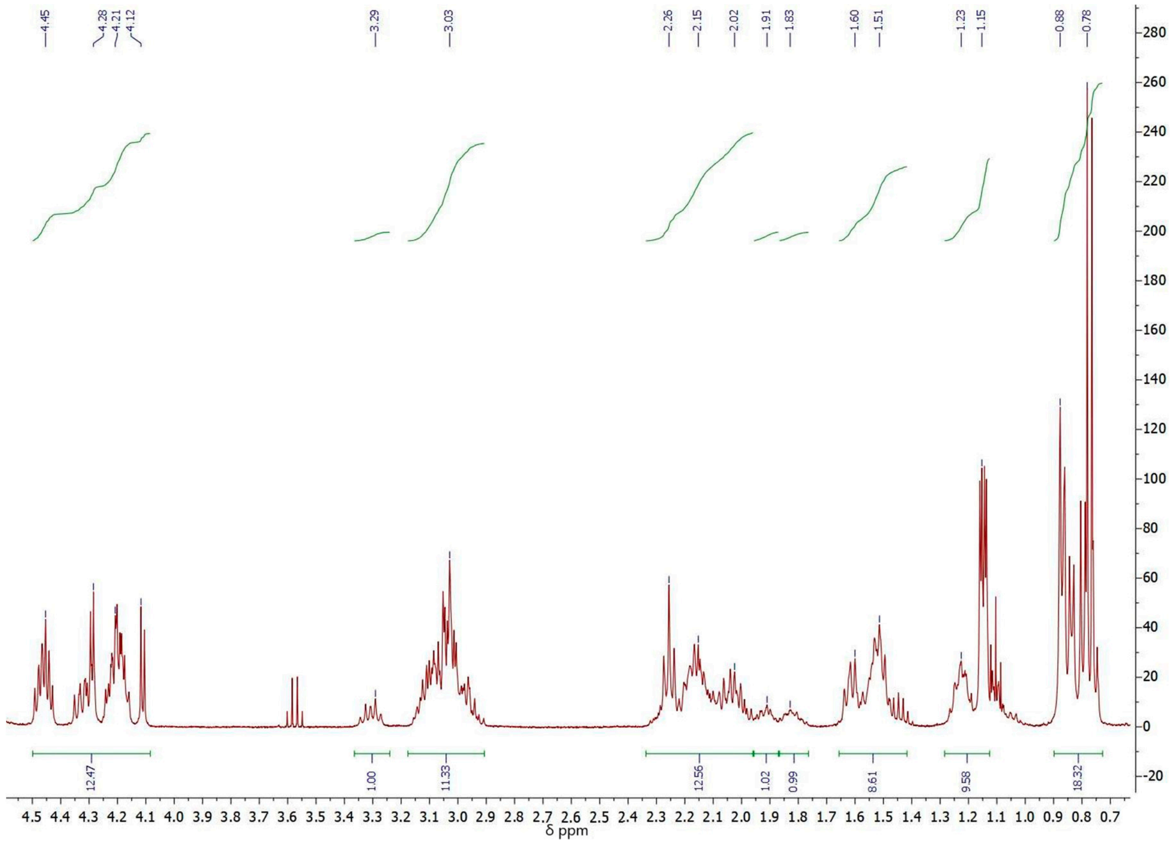

Figure S1. ^{1}H NMR spectrum of colistin (400 MHz, D_2O) δ 0.74-0.88 (m, 18H), 1.1-1.28 (m, 9,6H), 1.42-1.64(m, 8H), 1.83(m, 1H), 1.91 (m. 1H), 1.96-2.28(m, 12H), 2.92-3.15(m, 11H), 3,29(m, 1H), 4.10-4.50(m, 12H).

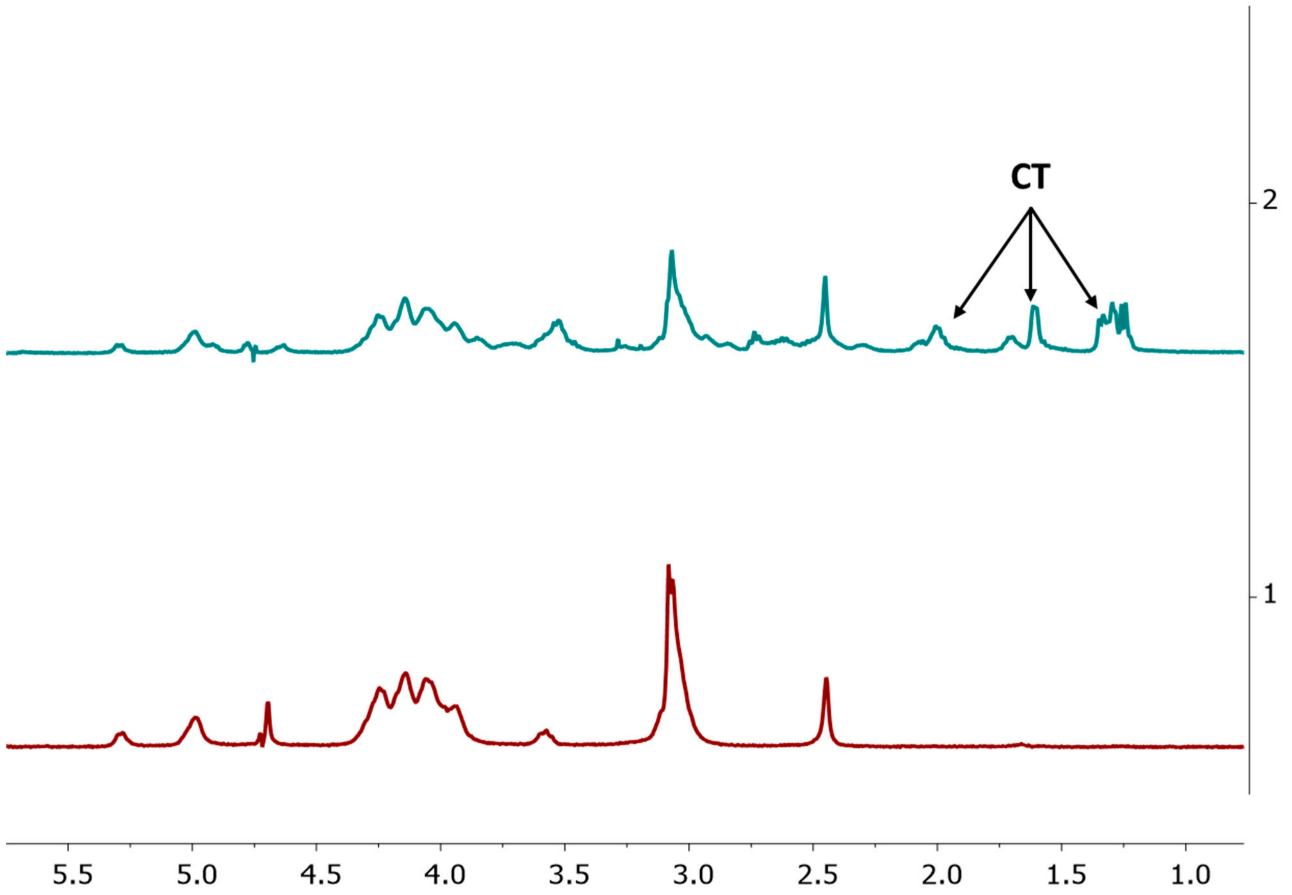

Figure S2. Stacked 1H NMR spectra of succinyl chitosan (1) and succinyl chitosan-colistin conjugate (2).

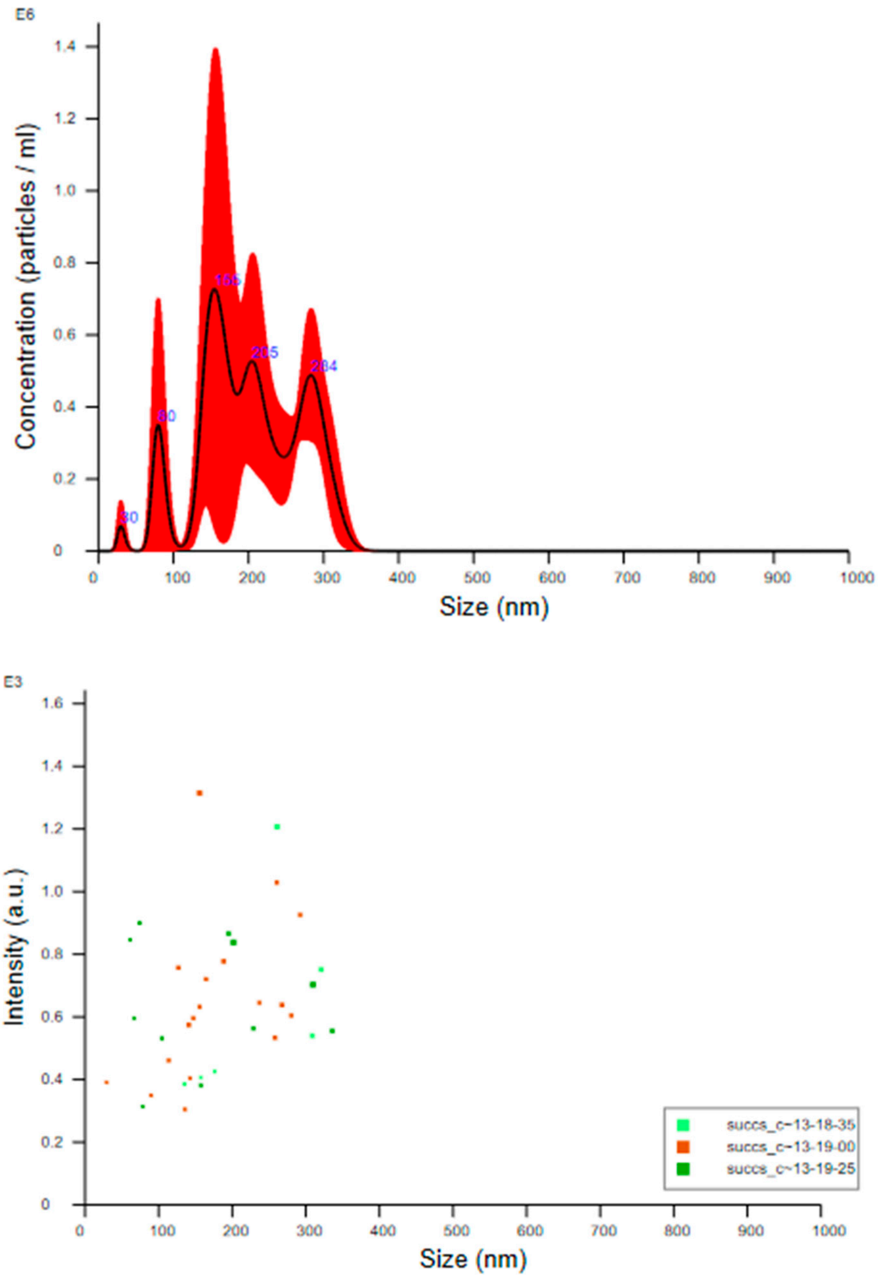

(a)

(b)

Figure S3. Mean particle size (a) and particle size distribution (b) of SucCS-CT-10 according to the NTA method.

Table S1. Viability of LPS-treated THP-1 cells after 24 h in vitro co-cultivation with SucCS-CT-10 conjugate. Data are presented as mean ± SD (n ≥ 6); the data are shown as the percentages of viable cells.

LPS stimulation only 94,5 ± 0,2			
**Concentration**	**SucCS**	**CT**	**SucCS-CT-10**
1000 μg/mL	93,6 ± 0,4	69,7 ± 3 ***	85 ± 2 ***
500 μg/mL	93,5± 0,5	86,1 ± 0,8 ***	93,6 ± 0,5
100 μg/mL	93,2 ± 0,7	93,2 ± 0,4	93,4 ± 0,6
10 μg/mL	93,3 ± 0,6	94,0 ± 0,4	93,8 ± 0,6
1 μg/mL	93,4 ± 0,6	93,8 ± 0,5	93,9 ± 0,5

*** – the differences with negative control (THP-1 cell without stimulation) were significant with $p < 0.001$, according to non-parametrical Mann–Whitney U test.
